# Biochemical characterization of extra- and intracellular endoxylanse from thermophilic bacterium *Caldicellulosiruptor kronotskyensis*

**DOI:** 10.1038/srep21672

**Published:** 2016-02-22

**Authors:** Xiaojing Jia, Weibo Qiao, Wenli Tian, Xiaowei Peng, Shuofu Mi, Hong Su, Yejun Han

**Affiliations:** 1 National Key Laboratory of Biochemical Engineering, Institute of Process Engineering, Chinese Academy of Sciences, Beijing 100190, China; 2 University of Chinese Academy of Sciences, Beijing 100049, China; 3 Institute of Apicultural Research, Chinese Academy of Agricultural Sciences, Beijing 100093, China

## Abstract

*Caldicellulosiruptor kronotskyensis* grows on lignocellulosic biomass by the catalysis of intrinsic glycoside hydrolase, and has potential application for consolidated bioprocessing. In current study, two predicted extra- (Xyn10A) and intracellular (Xyn10B) xylanase from *C. kronotskyensis* were comparatively characterized. Xyn10A and Xyn10B share GH10 catalytic domain with similarity of 41%, while the former contains two tandem N-terminus CBM22s. Xyn10A showed higher hydrolytic capability than Xyn10B on both beechwood xylan (BWX) and oat spelt xylan (OSX). Truncation mutation experiments revealed the importance of CBMs for hydrolytic activity, substrate binding and thermostability of Xyn10A.While the quantity of CBM was not directly related to bind and thermostability. Although CBM was considered to be crucial for substrate binding, Xyn10B and Xyn10A as well as truncations performed similar binding affinity to insoluble substrate OSX. Analysis of point mutation revealed similar key residues, Glu493, Glu601 and Trp658 for Xyn10A and Glu139, Glu247 and Trp305 for Xyn10B. Both Xyn10A and Xyn10B exhibited hydrolytic activity on the mechanical pretreated corncob. After pre-digested by Xyn10A or Xyn10B, the micropores inthe the mechanical pretreated corncob were observed, which enhanced the accessibility for cellulase. Compared with corncob hydrolyzed with cellulase alone, enhanced hydrolytic performance of was observed after pre-digestion by Xyn10A or Xyn10B.

Xylan, one of the major components of plant cell wall, represents an ideal renewable source for biorefinery^1^. Its complete degradation requires the synergism of several main and side chain-cleaving enzymes. Among xylanolytic enzymes, endo-β-1,4-xylanase (EC 3.2.1.8) randomly cleaves the β-1,4-xylosidic linkages of xylan backbone to produces xylo-oligosaccharides. Based on the amino acid sequence similarities and hydrophobic cluster analysis, endo-β-1,4-xylanases have been classified into glycoside hydrolase (GH) families 5, 8, 10, 11 and 43^2^. Xylanases play remarkable role in deconstruction of xylan containing lignocellulosic feedstock^3^. Thus they have gained wide interest in biofuel production, pulp bleaching, and feed industry.

It has been confirmed that some hemicellulases have complex modular constructions, in which catalytic domains (CD) and carbohydrate binding module (CBM) were connected by flexible linkers^4^. CBMs play an important role in biomass deconstruction by specifically recognizing target carbohydrates^5^. These non-catalytic carbohydrate-binding modules can promote the association of the enzyme with the carbohydrates, thereby enhance hydrolysis of polysaccharides. CBMs connected with GHs are generally recognized as binding domains, and considered to be valuable with their high affinities to bind hydrolytic substrates. In addition, previous publications have report that CBMs perform crucial functions on optimal temperatures and thermostability of hemicellulase^6^,^7^. Whereas, the fusion of CBM6s to *Bacillus halodurans* xylanase’s CD domains decreased its optimum temperature^8^.

The genus *Caldicellulosiruptor* is an extremely thermophilic bacterium, which can use xylan containing natural lignocellulosic biomass as carbonhydrate at high temperature (45–85 °C)^9^,^10^,^11^. Increasing attention has been attracted to the thermostable GHs encoded by *Caldicellulosiruptor* genome, and predicted extra- and intracellular xylanases belonging to different GHs (GH10 and GH11) were identified. Different from other congeneric species, *C. kronotskyensis* is the only one possessing all GH families collectively present in *Caldicellulosiruptor*^10^. Natural lignocellulosic biomass usually contain a range of heteropolysaccharides, *C. kronotskyensis* is more suitable for consolidated bioprocessing (CBP) for its wealth GH families. To understand the roles of xylanase for hydrolysis of xylan containing natural lignocellulosic biomass, the catalytic properties of predicted extra- and intracellular endoxylanases were investigated in present study. Two predicted extra- and intracellular GH10 xylanases from *C. kronotskyensis* were studied to explore catalytic module and mechanism for natural biomass degradation. The predicted extracellular xylanase (Xyn10A) Calkro_2008 has a GH10 catalytic domain (CD) and two tandem CBM22s. Whereas Calkro_2385 was predicted to be an intracellular xylanase (Xyn10B) with only GH10 CD, and the similarity of which to Xyn10A-CD was 41%. The catalytic properties of Xyn10B and Xyn10A, along with the corresponded truncations were biochemically characterized, and the functions of which for natural lignocellulosic biomass degradation were determined.

## Results

### Expression, purification and characterization of Xyn10A, Xyn10B and Xyn10A’s truncations

**Figure 1:**
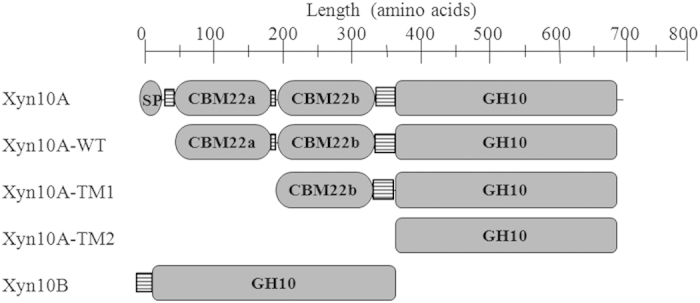
Modular architecture of Xyn10A, Xyn10B and truncations of Xyn10A. L1, L2: linkers; SP: signal peptide; GH10: catalytic domain of glycoside hydrolase family 10; CBM: carbohydrate binding module.

**Figure 2:**
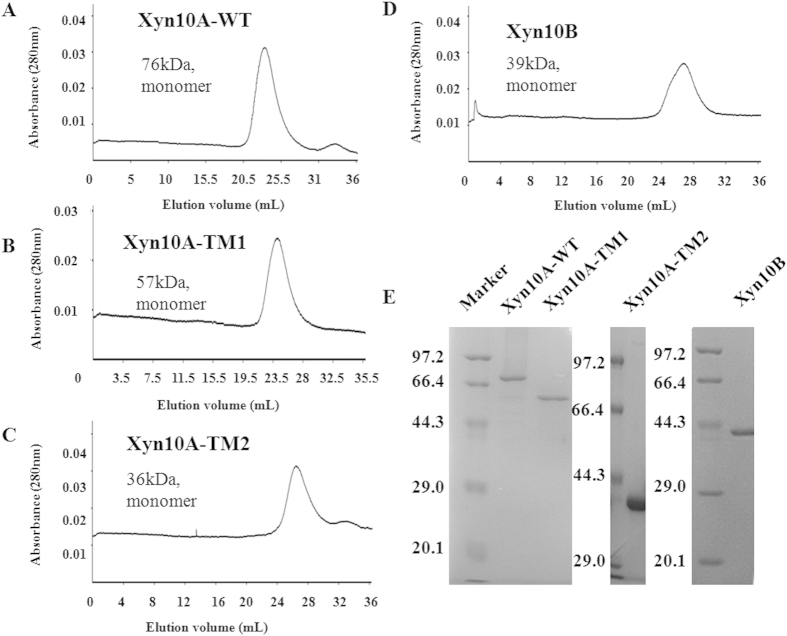
Purification and quaternary structure analysis of Xyn10A-WT, Xyn10A-TM1, Xyn10A-TM2 and Xyn10B. (**A**) Xyn10A-WT. (**B**) Xyn10A-TM1. (**C**) Xyn10A-TM2. (**D**) Xyn10B. (**E**) SDS-PAGE analysis of purified Xyn10A-WT, Xyn10A-TM1 and Xyn10A-TM2 and Xyn10B.

Through nucleotide sequence analysis, both *xyn*10A and *xyn*10B were defined to encode hypothetical GH10 endo-β-1,4-xylanase. Xyn10A and Xyn10B are respectively predicted to be extracellular and intracellular xylanase, as the former has a signal peptide at the N-terminal amino-acid polypeptides. For modular constitution, Xyn10A was composed of two N-terminally located tandem CBM22s and a C-terminally located GH10 CD (Fig. 1). The two tandem CBM22s (CBM22a and CBM22b) of Xyn10A are different, and the amino acids sequence identity is 25% (Fig. S1). The two linkers L1 (RPLSDISYYYIDNFTISDDGWYSAVPDLDLPS) and L2 (TNFYVDLFTLKVADKS) between CBM22s and CD (Fig. 1) shared sequence similarity of 44%. In contrast, there was only a GH10 CD on the polypeptide of Xyn10B (Fig. 1). To gain insight into the contribution of different domains to Xyn10A’s catalytic and binding activity, truncations of which were characterized. The wild type Xyn10A-WT, Xyn10B and Xyn10A-TM2 were expressed in *E. coli* BL21. Whereas, Xyn10A-TM1 was expressed in *E. coli* Rosetta, neither of the two CBM22s could be independently expressed with unknown reason. The predicted molecular weights of Xyn10A-WT, Xyn10A-TM1, Xyn10A-TM2 and Xyn10B were 74.7, 57.1, 38.3 and 39.9 kDa, respectively. Through size exclusion chromatography, quaternary structure of the four enzymes were further determined. All proteins appeared as single band, and the apparent molecular weights displayed by SDS-PAGE corresponded well to the predicted sizes, and suggested that all of the four proteins existed as monomers (Fig. 2).

**Figure 3:**
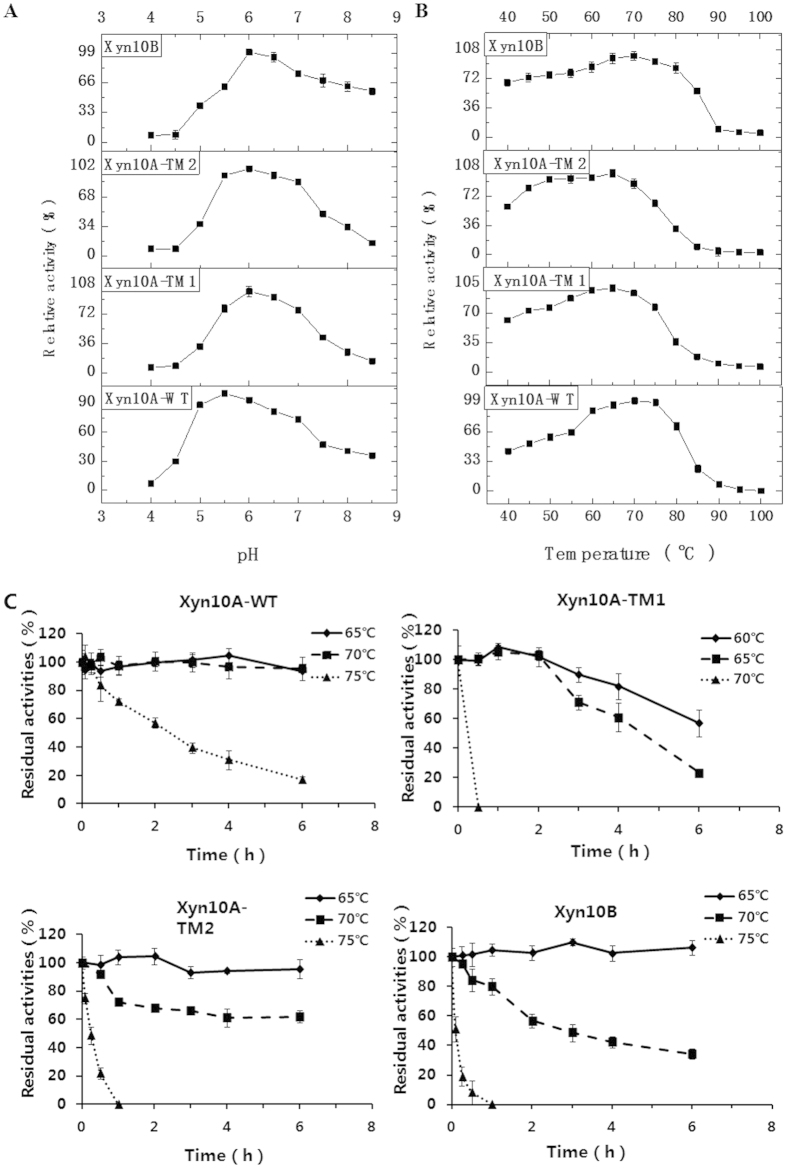
The property of pH, temperature, and thermostability for Xyn10A-WT, Xyn10A-TM1, Xyn10A-TM2 and Xyn10B. (**A**) Effect of pH on the activities. (**B**) Effect of temperature on the activities. (**C**) Effect of temperature on the thermostability.

Taking BWX as substrate, the optimal pH of Xyn10A-TM1, Xyn10A-TM2 and Xyn10B were 6.0, while 5.5 for Xyn10A-WT (Fig. 3A). The optimal temperature for both Xyn10A-WT and Xyn10B were 70 °C (Fig. 3B). For Xyn10A-TM1 and Xyn10A-TM2, the optimal temperature was 65 °C (Fig. 3B). The results of thermostability assay showed that Xyn10A-WT had the highest thermostability, and better than Xyn10B and Xyn10A’s truncations (Fig. 3C). Xyn10A-WT was stable at 70 °C after incubation for 6 h, and the half-life time (T1/2) was 2.3 h at 75 °C. The T1/2 of Xyn10A-TM1, Xyn10A-TM2 and Xyn10B at 75 °C were 0, 15 and 5 min, respectively. In light of this, the two mutations showed lower thermostability than the wild type, while the thermostability of Xyn10A-TM2 was better than that of Xyn10A-TM1 and Xyn10B. Interestingly, with the removal of CBM22a, the thermostability of Xyn10A-TM1 decreased significantly compared with Xyn10A-WT and Xyn10A-TM2.

As shown in Table 1, the specific activities of Xyn10A-WT, Xyn10A-TM1, Xyn10A-TM2 and Xyn10B on BWX were respectively 330.6 ± 3.5, 183.7 ± 12.2, 115.3 ± 1.1 and 122.1 ± 2.1 IU/mg. For OSX, the specific activities of Xyn10A-WT, Xyn10A-TM1, Xyn10A-TM2 and Xyn10B were 353.7 ± 8.0, 155.2 ± 3.5, 32.3 ± 6.7 and 118.6 ± 5.4 IU/mg, respectively (Table 1). It could be seen that Xyn10A-WT displayed the highest specific activity on BWX and OSX, Xyn10A-TM1 sharply halved, and Xyn10A-TM2 continuously decreased. Notably, Xyn10A-WT had lower activity (by 7%) on OSX than BWX, while Xyn10A-TM1 and Xyn10A-TM2 had much lower activity (by 15.5% and 72%) on OSX than BWX. With the removal of CBMs, the activity of Xyn10A-TM1and Xyn10A-TM2 showed higher activity on BWX than OSX. Meanwhile, the hydrolytic activity of Xyn10B was similar on BWX and OSX. Xyn10A-TM2 showed similar activity with Xyn10B on BWX, while much lower than Xyn10B on OSX (Table 1).

### Hydrolytic properties of Xyn10A, Xyn10B and Xyn10A’s truncations on BWX, OSX and XOS

**Figure 4:**
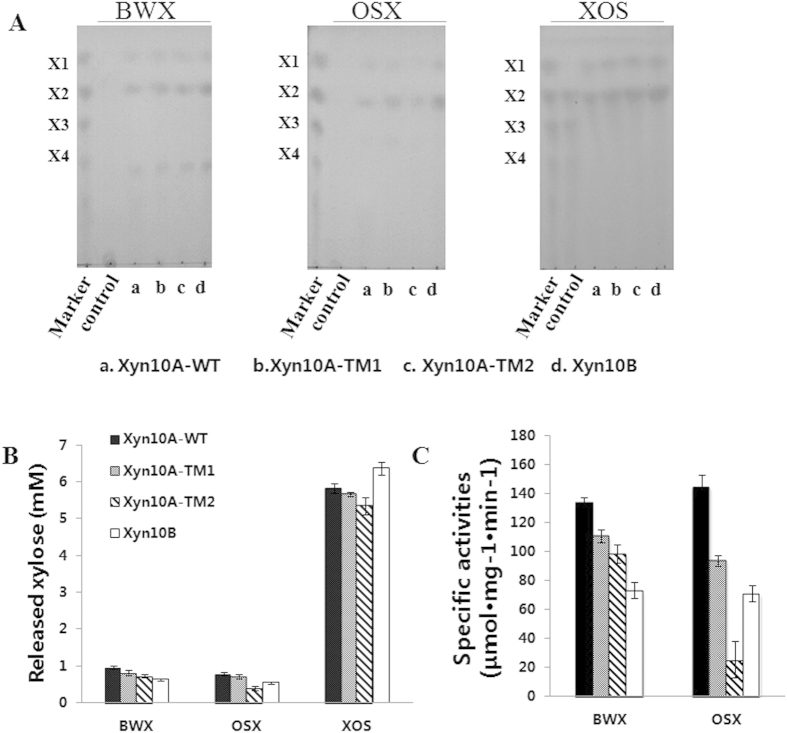
Hydrolytic patterns of Xyn10A-WT, Xyn10A-TM1, Xyn10A-TM2 and Xyn10B. (**A**) TLC analysis of the hydrolytic products of BWX, OSX and XOS. (**B**) HPLC analysis of the released xylose from BWX, OSX, and XOS. (**C**) Specific activity of Xyn10A-WT, Xyn10A-TM1, Xyn10A-TM2 and Xyn10B with BWX and XOS at 55 °C, pH 7.0. Each of purified Xyn10A-WT, Xyn10A-TM1, Xyn10A-TM2, and Xyn10B was incubated with BWX, OSX, and XOS at 55 °C for 16 h in 200 μl Tris buffer (pH 7.0, 50 mM Tris-HCl, 150 mM NaCl), respectively. The substrate XOS is consisted of xylobiose, xylotriose, xylotetraose, xylopentaose and other xylo-oligosaccharides. X1, xylose; X2, xylobiose; X3, xylotriose; X4, xylotetraose.

The hydrolytic model of the four enzymes on BWX, OSX and XOS were determined. As shown in Fig. 4, Xyn10A-WT, Xyn10A-TM1, Xyn10A-TM2 and Xyn10B released xylose, xylobiose and unknown oligosaccharides from BWX (Fig. 4A). While the productions of the enzymes on OSX and XOS were xylose and xylobiose (Fig. 4A). The results suggested that the main hydrolysis product of xylan or xylooligosaccharide hydrolyzed by the xylanases was xylobiose, and small quantity of xylose released by the β-1,4-xylosidic linkages random cleavage. The concentrations of released xylose from BWX by Xyn10A-WT, Xyn10A-TM1, Xyn10A-TM2 and Xyn10B were 0.96 ± 0.05, 0.82 ± 0.06, 0.72 ± 0.04, and 0.63 ± 0.02 mM, respectively. And those were 0.79 ± 0.03, 0.72 ± 0.05, 0.40 ± 0.06 and 0.55 ± 0.03 mM from OSX, and 5.83 ± 0.13, 5.66 ± 0.06, 5.35 ± 0.23 and 6.37 ± 0.17 mM from XOS, respectively (Fig. 4B). It’s interesting that the concentration of xylose released from XOS by Xyn10B was higher than that of Xyn10A-WT. In addition, at the same reaction condition (55 °C and pH 7.0), the specific activities of Xyn10A-WT and Xyn10B were 134.0 ± 3.5 and 73.2 ± 5.4 μmol mg^−1^ min^−1^ on BWX, and 144.5 ± 8.0 and 71.1 ± 5.4 μmol mg^−1^ min^−1^ on OSX, respectively (Fig. 4C).

### Binding properties of Xyn10A, Xyn10B and Xyn10A’s truncations on insoluble substrates

As shown in Fig. S2 52.0%, 65.4%, 60.9% and 49.6% of the proteins were left unbound when Xyn10A-WT, Xyn10A-TM1, Xyn10A-TM2 and Xyn10B were incubated respectively with insoluble OSX for 30 min at 4 °C. The deletion of CBM22 reduced the binding ability of Xyn10A with OSX. It was interesting that Xyn10B without CBM displayed higher affinity with OSX than Xyn10A. All of the four enzymes exhibited slight binding ability towards other insoluble substrates (Fig. S2). Xyn10A showed higher binding ability on xylan than the other materials, and displayed highest affinity among the four xylanases on xylan containing natural substrates.

### Hydrolytic properties of Xyn10A, Xyn10B and Xyn10A’s truncations on natural substrates and synergism with commercial cellulase

**Figure 5:**
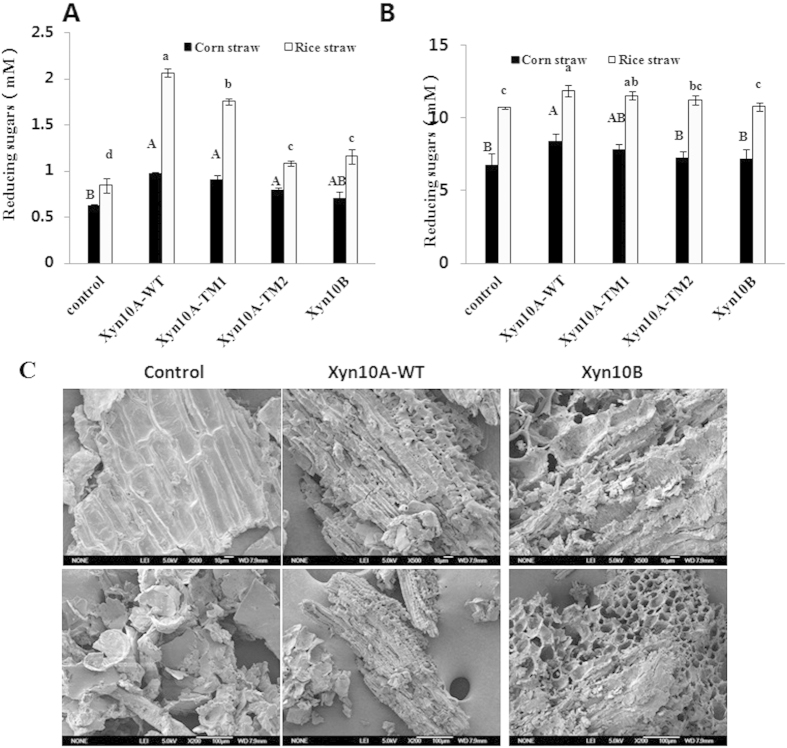
Pre-digestion of corn straw and rice straw with Xyn10A-WT, Xyn10A-TM1, Xyn10A-TM2 and Xyn10B for cellulase degradation. (**A**) Reducing sugars from corn straw or rice straw hydrolyzed by Xyn10A-WT, Xyn10A-TM1, Xyn10A-TM2 and Xyn10B; n = 3, Different uppercase or lowercase letters denote significant differences (P < 0.05). (**B**) Reducing sugars produced by Cellic CTec2 from corn straw or rice straw predigested with Xyn10A-WT, Xyn10A-TM1, Xyn10A-TM2 and Xyn10B; n = 3, Different uppercase or lowercase letters denote significant differences (P < 0.05). (**C**) FEG-SEM images of corncob particles hydrolyzed by Xyn10A-WT and Xyn10B; Control, Untreated corncob particles; Xyn10A-WT, Corncob particles digested by Xyn10A-WT; Xyn10B, Corncob particles digested by Xyn10B. Magnification of FEG-SEM images was 500 times.

To determine the hydrolytic activities on natural substrates, the xylanases was separately incubated with corn straw and rice straw under optimum condition. In Fig. 5A, products from the two substrates by enzymatic hydrolysis were observed, and the degradation of rice straw was better than that of corn straw. Significant differences of reducing sugars were found between the four xylanases compared to the control. As shown in Fig. 5B, after being pre-digested with the xylanases, the hydrolytic performance of the substrates was better than that of Cellic CTec2. Certainly, Xyn10A-WT performed the greatest capability on hydrolysis of natural lignocellulosic biomass among the four xylanases. With rice straw as the substrate, significant differences of reducing sugars were found between Xyn10A-WT, Xyn10A-TM1 and Xyn10A-TM2 and the control. After the enzymatic processing, pits and cracks were observed on the digested corncob, which verified the capability of single Xyn10A and Xyn10B could degrade natural corncob (Fig. 5C).

**Figure 6:**
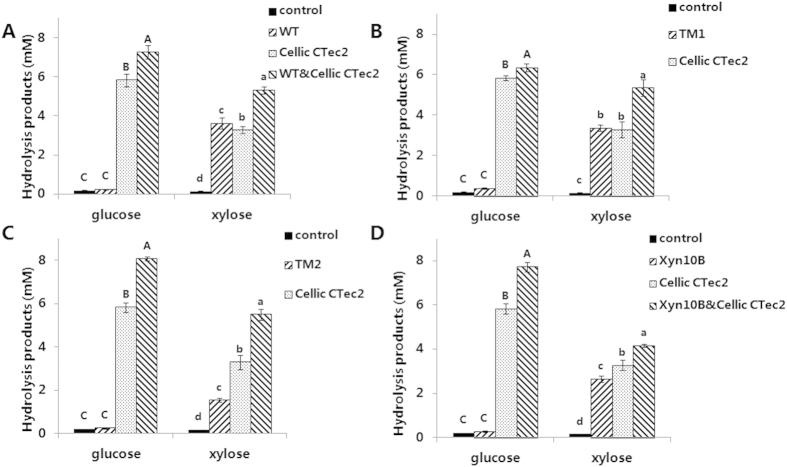
Hydrolytic properties and synergism with commercial cellulase Cellic CTec2 on corncob degradation. (**A**) Xyn10A-WT (WT). (**B**) Xyn10A-TM1 (TM1). (**C**) Xyn10A-TM2 (TM2). (**D**) Xyn10B. Each purified Xyn10A-WT, Xyn10A-TM1, Xyn10A-TM2 and Xyn10B 25 μg/ml was incubated individually or in combination with 15 IU/g Cellic CTec2 at 50 °C for 48 h. The reaction mixtures contained 2% (w/v) corncob particles in pH 5.5 Bis-Tris buffer (50 mM Bis-Tris, 150 mM NaCl). Hydrolytic products were determined by HPLC, glucose and xylose were used as standards.

The promotion of the xylanases for cellulase (Cellic CTec2, Novozymes) was analyzed in deconstruction of mechanically pretreated corncob at 50 °C. It was found that both single enzyme and enzyme cocktail exhibited positive effect in the enzymatic hydrolysis (Fig. 6). It is worthwhile to note that all of the four xylanases are active on mechanically pretreated corncob, especially Xyn10A-WT, Xyn10A-TM1 and Xyn10B. In the individual xylanase groups (Xyn10A-WT, Xyn10A-TM1, Xyn10A-TM2 and Xyn10B), the amounts of xylose generated were 3.63 ± 0.29, 3.36 ± 0.15, 1.54 ± 0.10 and 2.64 ± 0.14 mM, respectively. The amount of xylose produced by Xyn10A-WT was similar with that of Cellic CTec2, which is a cocktail composed of xylanase and cellulase. With the addition of xylanases (Xyn10A-WT, Xyn10A-TM1, Xyn10A-TM2 and Xyn10B) in hydrolysis, the respective amount of glucose increased by 24.7%, 8.8%, 38.5% and 32.7% compared with the individual Cellic CTec2 group (Fig. 6). The results indicated that Xyn10A-WT, Xyn10A-TM2 and Xyn10B promoted the hydrolysis of complicated natural substrate and improved monosaccharides production. The accessibility of Cellic CTec2 to cellulose might be increased with the addition of the four xylanases. In statistics analysis, the difference of monosaccharides production in corncob hydrolysis between with and without Xyn10A-WT, Xyn10A-TM2 or Xyn10B reached significant level.

### Site-directed mutagenesis to elucidate the catalytic residues

**Figure 7:**
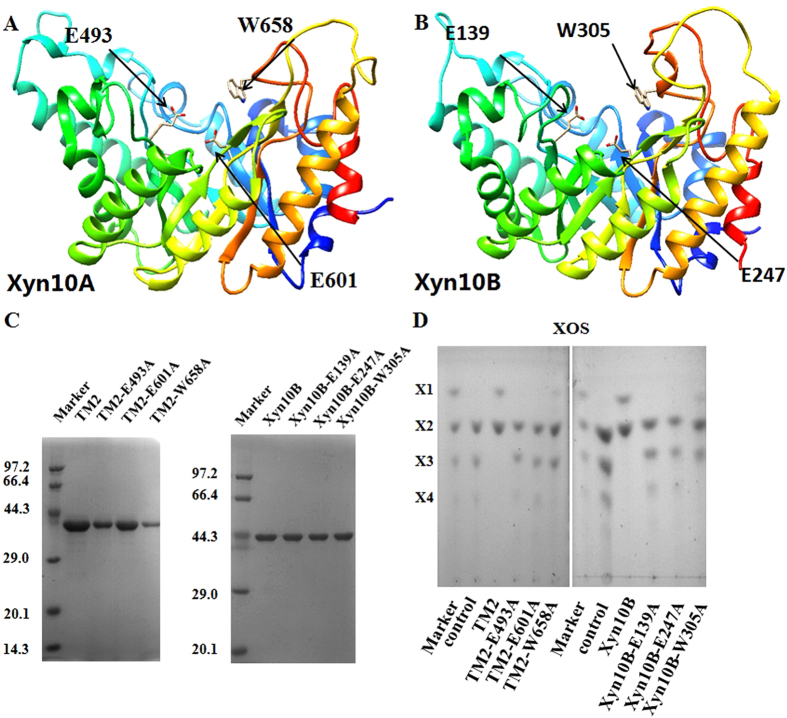
Key catalytic residues analysis of Xyn10A and Xyn10B through site-directed mutagenesis. The residues are Glu493, Glu601 and Trp658 for Xyn10A, and Glu139, Glu247 and Trp305 for Xyn10B. (**A**) Three-dimensional homology modeling of Xyn10A. (**B**) Three-dimensional homology modeling of Xyn10B. The three-dimensional homology models of Xyn10A and Xyn10B were constructed by the ModWeb server. The crucial residues Glu493, Glu601 and Trp658 for Xyn10A and Glu139, Glu247 and Trp305 for Xyn10B were marked by black arrows. (**C**) SDS-PAGE of the purified mutations. (**D**) Hydrolytic properties of the purified mutations on XOS. The reaction mixtures contained 25 μg/ml enzyme (Xyn10A-TM2, Xyn10B and their mutations) and 10 mg/ml XOS at 55 °C in citrate buffer (pH 6.0, 50 mM sodium citrate, 150 mM NaCl) for 6 h, the productions were analyzed by TLC. X1, xylose; X2, xylobiose; X3, xylotriose; X4, xylotetraose.

Both the catalytic nucleophile and catalytic proton donor of GH10 xylanases were glutamate (data from CAZY). Sequence alignment (computed on [http://www.genome.jp/tools/clustalw/] and depicted by ESPrit 3.0^12^ revealed conserved residues around catalytic region: Glu493, Glu601 and Trp658 of Xyn10A and Glu139, Glu247 and Trp305 of Xyn10B (Fig. S3). The nucleophile and proton donor in active site of Xyn10A and Xyn10B were Glu493 and Glu601 (Xyn10A), Glu247 and Trp305 (Xyn10B). Conserved residues Trp658 (Xyn10A) and Trp305 (Xyn10B), the aromatic residues of GH10 had been reported to have significant implication on substrate specificity^13^. These conserved residues were located in β6, β10 and η3 structure of Xyn10A and Xyn10B (Fig. S3). The three-dimensional homology models of Xyn10A and Xyn10B were constructed through ModWeb server (https://modbase.compbio.ucsf.edu/modweb/) (Fig. 7A,B).

By site-directed mutagenesis (The details were specified on Supplementary methods and data), the three conserved residues were respectively mutated to Alanine. All of the six recombinant mutations were analyzed by SDS-PAGE and the molecular weights corresponded well with the predicted sizes (Fig. 7C). In hydrolytic model analysis using XOS as substrates, Xyn10A-TM2-W658A and Xyn10B-W305A showed minor hydrolytic activity with small amount of xylose as product. No xylose was observed when XOS was hydrolyzed with Xyn10A-TM2-E493A, -E601A, or Xyn10B-E139A, -E247A (Fig. 7D). The specific activities of Xyn10A-TM2-W658A and Xyn10B-W305A on BWX were 1.7 ± 0.2 and 0.7 ± 0.3 IU/mg, respectively (Table 2). The binding affinity of the six mutations on OSX was similar to that of the native enzymes. The results indicated that the three residues of Xyn10A and Xyn10B were vital for catalysis, while the binding property on insoluble substrates was not affected.

## Discussion

The thermophilic cellulolytic bacteria of genus *Caldicellulosiruptor* have the ability to grow on xylan containing natural lignocellulosic biomass without pretreatment. Different from other congenic species, *C. kronotskyensis* is the only one possessing all cellulase and hemicellulase GH families collectively present in *Caldicellulosiruptor*. With abundant genes of different GH families present in genome, *C. kronotskyensis* is a potential candidate for consolidated bioprocessing of lignocellulosic biomass. Xyn10A was a secreted GH10 xylanase with two CBM22s flanked on its N-terminus, whereas Xyn10B was an intracellular xylanase with just GH10 catalytic module. Generally, extracellular enzymes would degrade high polymers into low molecular polymers, which could readily enter the cell via specific ABC sugar transporters^14^. The short polymers would be further hydrolyzed to xylose by intracellular enzymes such as xylanases and β-xylosidases. Xyn10A showed highest hydrolytic activity among the four xylanases, and the specific activity on OSX was higher than BWX. While the hydrolytic activity of Xyn10A-TM1, Xyn10A-TM2, and Xyn10B on OSX was lower than BWX. The results indicated that the different substrate preference of extra- (Xyn10A) and intracellular xylanase (Xyn10B), Xyn10A prefer insoluble substrate than the soluble one. While with the removal of CBM22, Xyn10A prefer soluble substrate than insoluble, the hydrolytic property was similar with Xyn10B. The specific activity on BWX of Xyn10A-TM2 was higher than Xyn10B, while the activity of Xyn10A-TM2 on OSX was lower than Xyn10B. With XOS was substrate, the xylose produced from Xyn10B was higher than Xyn10A and the truncations.

Corn stover, rice straw and corncob, as abundant agricultural by-products with low commercial value, are potential lignocellulose-rich energy resources. The plant cell wall is a complex of biopolymers consisted primarily of cellulose, hemicellulose and lignin in various contents^3^. Among these polysaccharides, xylan is usually considered to be the limiting factor during enzymatic hydrolysis of crystalline cellulose in the presence of xylan content^15^. Several reports had shown the positive effect of the synergistic interaction between xylanases and cellulases on the hydrolysis of different polysaccharide compounds^16^,^17^,^18^. All of the four xylanases displayed hydrolytic activity on natural substrates, and the promoted the following hydrolysis by Cellic CTec2 cellulase. The competitive relationship between these purified xylanases with xylanase from Cellic CTec2 showed the degree of synergy improvement would reduce with the increase of amount of xylanase. Among the four xylanases, Xyn10A was the best enzyme for hydrolysis. Compared with the individual Cellic CTec2 treatment, the addition of xylanases (Xyn10A-WT, Xyn10A-TM1, Xyn10A-TM2 and Xyn10B) could accelerate the deconstruction of corncob and improve the production of monosaccharides. Considering the preference of intracellular xylanase Xyn10B for short xylo-oligosaccharides, the extracellular xylanase Xyn10A more preferentially hydrolyzes long and branched polysaccharides^19^. The intracellular xylanse Xyn10B also exhibited efficient hydrolytic capability on both insoluble xylan and natural lignocellulose. These xylanases thus have great potentials for the bioconversion of complex polysaccharides to soluble saccharides. The pre-digestion of biomass with enzymes at high temperature for efficient hydrolysis of biomass has been reported previously^20^.Thermostable xylanases have significant advantages for pre-digestion of natural lignocellulosic biomass over their mesophilic counterparts.

Xyn10A, Xyn10B and Xyn10A’s truncations retained considerable activities in extreme thermal environment. Especially the wild type Xyn10A-WT, which was stable at 70 °C after incubating for 6 h and had a half-life at 75 °C of over 2 h. The sequential deletion of CBM22s from the wild type Xyn10A had a marked impact on the thermostability. The deletion of CBM lowered the thermostability of the wild type, similar results had been reported in other literature^21^. Interestingly, the thermostability of Xyn10A-TM1 with CBM22b was lower than that of Xyn10A-TM2 without CBM. The phenomenon was also reported by Araki *et al.* and found that the deletion of CBM22 improved thermostability of Xyn10B from *Clostridium stercorarium*, the mechanism might be eliminating the prevention of CBM22 on thermal refolding^22^. It had been reported that the protein thermostability was affected by disulfide bond, hydrophobic interaction, ionic bond and aromatic-aromatic interaction^23^,^24^,^25^. The disulfide bond of Xyn10A-WT and its truncations was predicted by using website (http://bioinformatics.bc.edu/clotelab/DiANNA/). The numbers of predicted bonds of Xyn10A-WT, Xyn10A-TM1and Xyn10A-TM2 are 2, 0 and 0, suggesting that disulfide bond is one of the major factors for the higher thermostability of Xyn10A-WT than its truncations^26^. In addition, charged surface residues also affect protein stability for the resistance to denaturation and assistance to enzyme folding^26^,^27^. The change of surface amino acid residues, addition or deletion of residues would probably affect the surface charge–charge interactions of native proteins, thereby impact the protein stability^23^.

In current study, it was obvious that the deletion of CBM22s reduced the activities of Xyn10A, indicating that CBM22s play a crucial role on the activity of Xyn10A. The removal of CBM22s reduced the activity of Xyn10A on OSX significantly, suggesting CBM22s played a crucial role on hydrolysis of insoluble substrate than soluble substrate. Previous study also demonstrated that CBM improves xylanase’s activity towards insoluble xylan^28^. In most case, CBMs connected with GHs are generally recognized as binding domains, and considered to be valuable with their high affinities to bind substrates^29^. Interestingly, deletion of CBM22 reduced the binding ability of Xyn10A with OSX slightly. Meanwhile, the binding experiments also found that the GH10 domain (Xyn10B and Xyn10A-TM2) CD possesses the binding affinity.

The crucial conserved residues for catalysis were predicted by sequence alignment by using *C. bescii* Cbxyn10b [PDB: 4L4O] as template. The Glu493/Glu139 in β6, Glu601/Glu247 in β10 and Trp658/Trp305 in η3 were highly conserved in Xyn10A and Xyn10B, respectively (Fig. S3). Glutamate was normally considered as the catalytic nucleophile and catalytic proton donor in GH10 proteins. Xyn10A and Xyn10B possessed the typical (β/α)_8_ barrel of GH10, and these three residues were located near the catalytic center of protein structures (Fig. 7A,B). Glu493, Glu601and Trp658 for Xyn10A and Glu139, Glu247 and Trp305 for Xyn10B were then replaced by Alanine. Mutations in Glu493 and Glu601 of Xyn10A and Glu139 and Glu247 of Xyn10B instantly abolished hydrolytic activities on xylan substrates. The mutations in Trp658 of Xyn10A and Trp305 of Xyn10B also demonstrated their importance for catalytic activity on xylan. The binding affinities of the six mutations on OSX were detected as well.

In conclusion, two distinct GH10 xylanases from *C.kronotskyensis*, extra-xylanase Xyn10A and intra-xylanase Xyn10B were comparatively characterized in present study. Xyn10A displayed higher activity on insoluble highly polymerized xylan. Truncation mutants suggested the importance of CBM22s for hydrolytic activity and thermostability of Xyn10A. With the removal of CBM22s, the hydrolytic activity of Xyn10A truncations decreased gradually. It is worth noting that Xyn10A-TM1 with CBM22b displayed lower thermostability than Xyn10A-TM2 without CBM22. Xyn10A-TM2 and Xyn10B shared similar modular architecture, thermostability and key catalytic residues, while the latter showed higher hydrolytic activity on OSX. Although predicted as intracellular endoxylanase, while Xyn10B showed binding property on OSX. The affinity of Xyn10A-TM2 and Xyn10B on OSX suggested that the GH10 catalysis domain play an important role for substrate binding. Both Xyn10A and Xyn10B showed hydrolytic activity toward xylan-containing natural lignicellulosic biomass. Holes are observed on corncob by processing with Xyn10A-WT or Xyn10B, and the accessibility substrate was therefore increased.

## Methods

### Materials

Beech wood xylan (BWX, Sigma, USA), oat-spelt xylan (OSX, Shanghai Hualan Chemical Technology Co., Ltd, China), xylooligosaccharides (XOS, Longlive Bio-Technology Co., China), Avicel (Kepujia Reagent Co., China), corncob particles, steam explosion pretreated corn straw (SETCS), grinded corn straw and grinded rice straw were used for hydrolysis experiments. SETCS was provided by Chen lab from Institute of Process Engineering, Chinese Academy of Sciences. Corncob particles, grinded corn straw and grinded rice straw were products treated by mechanical processing by pulverizer (400Y, Kepujia Reagent Co., China). The xylan content (w/w, %) of rice straw, corn straw and corncob particles and SETCS are 22, 17, 36, and 0%. *Escherichia coli* Top10 (TianGen, China) was used for gene cloning and plasmid maintenance. *E. coli* BL21 (DE3) and Rosetta (DE3) were applied for gene expression. Plasmid pET-28b (+) (Novagen, USA) was used for recombinant plasmid construction and gene expression. *C. kronotskyensis* 2002 (DSM 18902) was purchased from DSMZ (Braunschweig, Germany). All of the other reagents and chemicals stated were of analytical grade.

### Gene cloning, expression, and protein purification

The complete genome sequence of *C.kronotskyensis*^30^ was analyzed on http://rast.nmpdr.org/rast.cgi^31^, and the genes Calkro_2008 (*xyn*10A) and Calkro_2385 (*xyn*10B) were annotated as GH10 xylanases^30^. Xyn10A was a polypeptide with three modules: a GH10 and a tandem CBM22s (CBM22a and CBM22b). For Xyn10B, it contains only one GH10 CD. Signal peptides at the N terminus of each protein were predicted by Signal-3L (http://www.csbio.sjtu.edu.cn/bioinf/Signal-3L/)^32^. The coding sequences for Xyn10A wild type (Xyn10A-WT), Xyn10B and the truncations (Xyn10A-TM1: Xyn10A-CD-CBM22b, Xyn10A-TM2: Xyn10A-CD) of Xyn10A (shown in Fig. 1) were amplified from the genomic DNA of *C. kronotskyensis* by PCR using Pfu DNA polymerase (TianGen, China). The extraction of genomic DNA was performed by using TIANamp Bacteria DNA Kit (TianGen, China). The primers for cloning the genes were listed in Table S1. The purified PCR products of *WT*, *TM1*, *TM2* of *Xyn10A* were separately treated with 0.5 IU T4 DNA polymerase (Takara, China), and then cloned into the pET-28b Ek/LIC vector. As for *Xyn10B*, the PCR product was purified and digested by *Nde* I/*Xho* I (Takara, China), and then inserted into pET-28b vector with same digestion. The recombinant plasmids were transformed into *E. coli* Top10 competent cells by heat shock, respectively. Colonies were screened on lysogeny broth (LB) agar plates supplemented with kanamycin (50 μg/ml) at 37 °C for 12 h. After identification by colony PCR and sequencing validation, positive recombinant plasmids were extracted by using TIAN prep Mini Plasmid Kit (TianGen).

The recombinant plasmids of Xyn10A-WT, Xyn10A-TM2 and Xyn10B were transformed individually into *E. coli* BL21 (DE3). Recombinant plasmid of Xyn10A-TM1 was transformed into *E. coli* Rosetta (DE3). After 16 h incubation at 37 °C, single colonies were picked and inoculated individually into LB medium supplemented with the same antibiotics. Those positive colonies were cultured overnight at 37 °C with a shaking frequency of 220 rpm/min. Each culture was diluted 100-fold in fresh LB (1 liter) containing antibiotic and grown at 37 °C with shaking (220 rpm) until the optical density reached 0.6 at 600 nm. A final concentration of 0.1 mM IPTG was added to each culture and then incubated at the same conditions for another 5 h. Cells were collected by centrifugation (3000 g) for 20 min at 4 °C and resuspended in lysis buffer (50 mM Tris-HCl, pH 7.5, 300 mM NaCl). The resuspended cell pellets were lysed by sonication and centrifuged at 13,000 g for 30 min at 4 °C. All supernatants were incubated with His-Tag Ni-affinity resin, and then fusion proteins were eluted using elution buffer (500 mM imidazole, 50 mM Tris-HCl, pH 7.5, 300 mM NaCl). To purify and determine the quaternary structure of recombinant proteins, eluted proteins were further analyzed by size exclusion chromatography. After gel filtration, the purity of the target proteins was analyzed by sodium dodecyl sulfate-polycrymide gel electrophoresis (SDS-PAGE). Concentrations of the purified proteins were measured at 595 nm by the Coomassie Brilliant Blue G250 binding method using bovine serum albumin (BSA) as standard.

### Biochemical characterization of Xyn10A and Xyn10B

The properties of optimal pH, temperature, and thermostability for Xyn10A WT, TM1, TM2 and Xyn10B were measured in citrate buffer (50 mM sodium citrate, 150 mM NaCl) or phosphate buffer (50 mM phosphate, 150 mM NaCl) with 2.5 mg/ml BWX as substrate. The optimum temperature for these xylanases was determined by measuring the activity at a temperature range of 40–100 °C in pH 6.0. The optimum pH was assayed in different buffers (citrate buffer pH 4.0–6.5; phosphate buffer pH 7.0–8.5) at optimum temperatures (Xyn10A-WT and Xyn10B: 70 °C; Xyn10A-TM1 and Xyn10A-TM2: 65 °C). To determine the thermostability, purified enzymes were incubated in citrate buffer pH 6.0 for different periods (0, 0.5, 1, 2, 3, 4, 6 h), and the residual activities were measured under optimal conditions. The temperature applied for Xyn10A-WT, Xyn10A-TM2 and Xyn10B were 65, 70, and 75 °C and 60, 65, and 70 °C for Xyn10A-TM1. The specific activity of Xyn10A-WT, Xyn10A-TM1, Xyn10A-TM2 and Xyn10B on BWX (2.5 mg/ml) and OSX (10 mg/ml) were determined at optimal pH and temperature. The reaction time was 2 min, and the relation of reducing sugar released versus time was linear in a 10-min assay. Under the optimized conditions, the amount of enzyme which catalyzed the production of 1 μmol reducing sugars per minute was defined as one unit. The reducing sugars released were measured by PAHBAH method by using xylose as standard^33^.

### Hydrolytic properties of Xyn10A and Xyn10B on BWX, OSX and XOS

In the hydrolysis, the enzyme loading was 2.5 mg/g substrate. The reaction mixtures containing 10 mg/ml BWX, OSX or XOS in 200 μl Tris buffer (50 mM Tris–HCl, 150 mM NaCl; pH 7.0) were incubated at 55 °C for 16 h, and discontinued by boiling water bath for 10 min. The samples were then centrifuged at 13,000 g for 10 min and filtered with 0.22 μm organic membrane filters (Millipore, USA), and analyzed with thin-layer chromatography (TLC) and high performance liquid chromatography (HPLC)^34^.

### Binding experiment of Xyn10A and Xyn10B on insoluble substrates

The substrates (with 200 mesh sieve screening), OSX, Avicel, corncob particles, steam explosion pretreated corn straw, grinded corn straw and grinded rice straw, used for affinity experiment were washed with water for three times, dried and mixed with single enzyme (Xyn10A-WT, Xyn10A-TM1, Xyn10A-TM2 or Xyn10B) separately in citrate buffer (50 mM sodium citrate, 150 mM NaCl pH 6.0) with a final concentration of 1% (w/v). The enzyme-substrate mixtures were incubated in a vertical mixing apparatus at 4 °C for 30 min, and the rest unbound enzymes in the supernatant after centrifugation were measured at 595 nm by Bradford method. All binding studies were performed in triplicates and BSA was incubated with each substrate as a control.

### Hydrolytic properties of Xyn10A and Xyn10B on natural substrates and synergism with cellulase

Natural substrates (corn straw, rice straw and corncob) were preprocessed by mechanical comminution and then washed with distilled water. Each enzyme Xyn10A-WT, Xyn10A-TM1, Xyn10A-TM2, Xyn10B (final concentration: 1.25 mg/g substrate ) was separately incubated with corn straw, rice straw and corncob particles (final concentration: 2%, w/v) in citrate buffer (50 mM sodium citrate, 150 mM NaCl; pH 6.0) at 50 °C for 48 h, and the reaction was stopped by boiling at 100 °C for 10 min. The reducing sugar produced in the reactions was measured by PAHBAH method. Statistical analysis was conducted by using SPSS ver. 17.0 (SPSS Inc., Chicago, IL, USA) software package. Significance was accepted at P < 0.05. Statistical differences were analyzed by one-way ANOVA followed by the LSD test. The microscopic structure modification insight into the corncob digested by Xyn10A-WT and Xyn10B were observed by FEG-SEM (Field Emission Gun Scanning Electron Microscopy) JSM-6700F (JEOL, Japan).

The natural substrates pre-processed by xylanases were washed three times with distilled water, the pretreated corn straw and rice straw were further hydrolyzed by commercial cellulase (Cellic CTec2, Novozymes, final concentration: 15 IU/g substrate) at 50 °C and pH 5.0 (optimum conditions for Cellic CTec2) for 24 h, and the reducing sugars were determined thereafter.

To explore the effect of xylanase on commercial cellulase for corncob hydrolysis, the xylanases Xyn10A-WT, Xyn10A-TM1, Xyn10A-TM2, and Xyn10B (final concentration: 1.25 mg/g corncob particles) were incubated separately or in combination with Cellic CTec2 (final concentration: 15 IU/g corncob particles), and the reaction mixtures containing corncob particles (final concentration, 2%, w/v) in Bis-Tris buffer (50 mM Bis-Tris, 150 mM NaCl pH 5.5) were incubated at 50 °C for 48 h. After the reaction was stopped by incubating in boiling water bath for 10 min, the samples were centrifuged at 13,000 g for 10 min and filtered with 0.22 μm organic membrane filters. The hydrolytic products were assayed by HPLC method by using glucose or xylose as standard. Statistical differences were analyzed by one-way ANOVA followed by the LSD test.

### Site-directed mutagenesis for key residue analysis

Mutagenic primers were listed in Table S1. Mutations were performed by PCR using plasmids Xyn10A-TM2- or Xyn10B-pET-28b as template with KOD-plus DNA polymerase (TOYOBO, Japan). The purified PCR products were then incubated with *Dpn* I (Takara, China) at 37 °C for 4 h, and the digested products without parent plasmid were transformed into *E. coli* Top10 by heat shock method. Colonies were screened on LB agar plates with kanamycin at 37 °C for 12 h, and positive mutations were sequenced to ensure appropriate mutations. Expression and purification of the mutations were similar with that of wild type Xyn10A and Xyn10B. To determine the activity of mutations, each purified enzyme (final concentration: 2.5 mg/g XOS) was incubated with 10 mg/ml XOS at 55 °C for 6 h, respectively. The hydrolytic products were assayed by using TLC method as described above. The specific activity of each mutation was measured by PAHBAH method with BWX as substrate.

## Additional Information

**How to cite this article**: Jia, X. *et al.* Biochemical characterization of extra- and intracellular endoxylanse from thermophilic bacterium *Caldicellulosiruptor kronotskyensis*. *Sci. Rep.* **6**, 21672; doi: 10.1038/srep21672 (2016).

## Supplementary Material

Supplementary Information

## Figures and Tables

**Table 1:** Specific activities of Xyn10A-WT, Xyn10A-TM1, Xyn10A-TM2 and Xyn10B with BWX and OSX.

	Specific activities^a^ (IU/mg)	
	BWX	OSX
Xyn10A-WT	330.6 ± 3.5	353.7 ± 8.0
Xyn10A-TM1	183.7 ± 12.2	155.2 ± 3.5
Xyn10A-TM2	115.3 ± 1.1	32.3 ± 6.7
Xyn10B	122.1 ± 2.1	118.6 ± 5.4

^a^The specific activity of each enzyme was determined under optimal conditions with BWX and OSX as substrates.

**Table 2:** Specific activities of the mutations of Xyn10A-TM2 and Xyn10B with BWX as substrate^a^.

Mutagenesis	Specific activities(IU/mg)	Desiredmutation
Xyn10A-TM2-E493A	0	Glu493Ala
Xyn10A-TM2-E601A	0	Glu601Ala
Xyn10A-TM2-W658A	1.7 ± 0.2	Trp658Ala
Xyn10B-E139A	0	Glu139Ala
Xyn10B-E247A	0	Glu247Ala
Xyn10B-W305A	0.7 ± 0.3	Trp305Ala

^a^The activities of Xyn10A-TM2 and Xyn10B were respectively 115.3 and 122.1 (IU/mg).
