# Supplementary material for: Biochemical characterization of extra- and intracellular endoxylanse from thermophilic bacterium Caldicellulosiruptor kronotskyensis
Source: Sci Rep. 2016 Feb 22;6:21672. doi: 10.1038/srep21672 (PMC4761950; doi:10.1038/srep21672)
Supplement: Supplementary Information [file srep21672-s1.pdf]

Biochemical characterization of extra- and intracellular endoxylanse from thermophilic bacterium *Caldicellulosiruptor kronotskyensis*

Xiaojing Jia[1,2¶], Weibo Qiao[1¶], Wenli Tian[3], Xiaowei Peng[1], Shuofu Mi[1], Hong Su[1,2], Yejun Han[1]*

[1]National Key Laboratory of Biochemical Engineering, Institute of Process Engineering, Chinese Academy of Sciences, Beijing 100190, China;

[2]University of Chinese Academy of Sciences, Beijing 100049, China;

[3]Institute of Apicultural Research, Chinese Academy of Agricultural Sciences, Beijing 100093, China

* Corresponding author:

Email: yejunhan09@gmail.com

¶ These authors contributed equally to this work.

Supporting Information

Figure S1. Sequence alignment of CBM22a and CBM22b of Xyn10A. Sequence alignment was computed on http://www.genome.jp/tools/clustalw/ and depicted by ESPrit 3.0.

Figure S2. Binding ability of Xyn10A-WT, Xyn10A-TM1, Xyn10A-TM2 and Xyn10B on different insoluble substrates. Each recombinant protein was mixed with the substrates in pH 6.0 citrate buffer (50 mM sodium citrate, 150 mM NaCl) at a final concentration of 1% (w/v) and incubated in a vertical mixing apparatus at 4 ℃ for 30 min. After treatment, the enzyme-substrate mixtures were centrifuged, and the unbound enzymes in the supernatant were measured at 595 nm by the Bradford method. SETCS, steam explosion pretreated corn straw.

Figure S3. Sequence alignment of Xyn10A and Xyn10B with other GH10 xylanases. Sequence alignment was computed on http://www.genome.jp/tools/clustalw/ and depicted by ESPrit 3.0. The residues Glu493, Glu601and Trp658 for Xyn10A and Glu139, Glu247 and Trp305 for Xyn10B are marked by black arrows. The alignment includes the xylanases from *C. bessi* [PDB: 4L4O], *P. barcinonensis* [PDB: 3EMC_A], *G. stearothermophilus* [PDB: 2Q8X], *C. stercorarium* [PDB: 2DEP] and *C. japonicus* [PDB: 1US3].

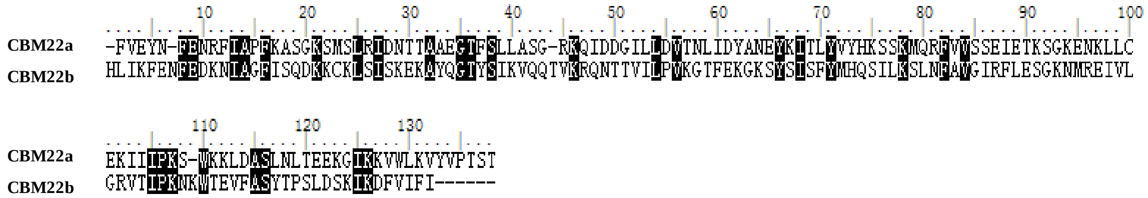
10 20 30 40 50 60 70 80 90 100
CBM22a -FVEYN-FENRFIAPFKASGKSMSLRIDNTTAAEGTFSLLASG-RKQIDDGILLDVTNLIDYANEYKITLYVYHKSSKMQRFVVSSEIETKSGKENKLLC
CBM22b HLIKFENFEDKNIAGFISQDKKCKLSISKEKAYQGTYSIKVQQTVKRQNTTVILPVKGTFEKGKSYSISFYMHQSILKSLNFAVGIRFLESGKNMREIVL
110 120 130
CBM22a EKIIIPKS-WKKLDASLNLTEEKGIKKVWLKVYVPTST
CBM22b GRVTIPKNKWTEVFASYTPSLDSKIKDFVIFI------

Figure. S1

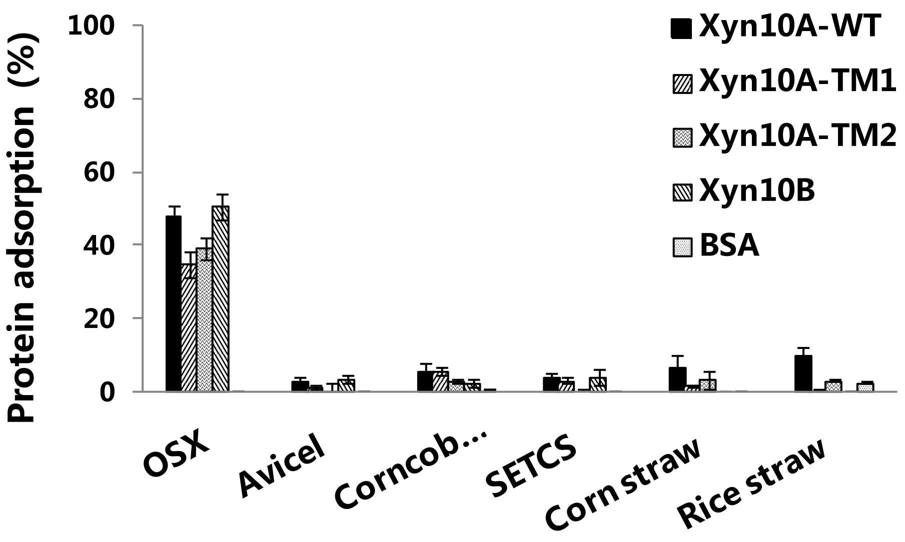
Protein adsorption (%)
100
80
60
40
20
0
Xyn10A-WT
Xyn10A-TM1
Xyn10A-TM2
Xyn10B
BSA
OSX
Avicel
Corncob…
SETCS
Corn straw
Rice straw

Figure.S2

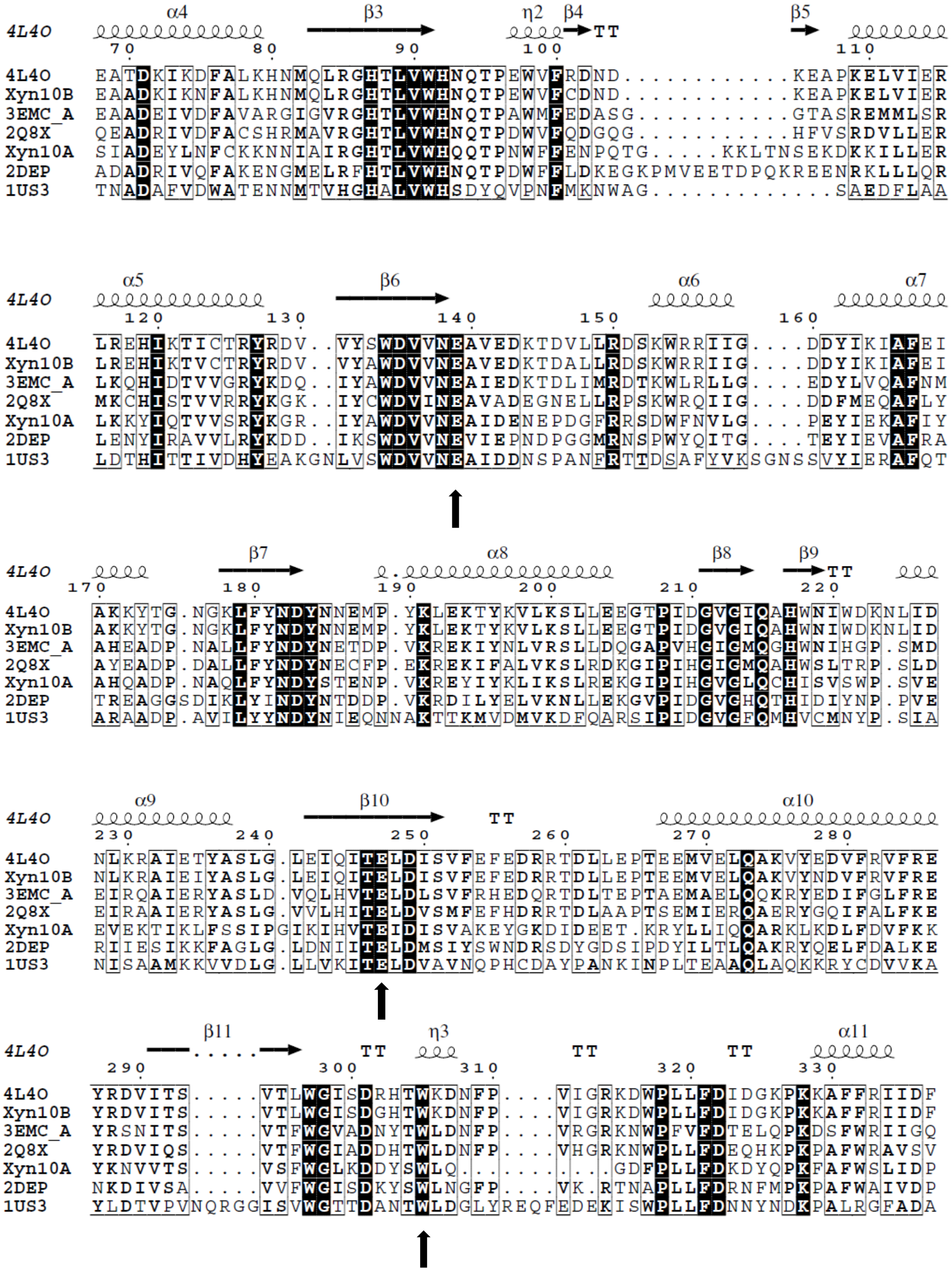

56

57 **Figure.S3**

58

Table S1. Primers used in this study

Gene use and name	Orientation	Sequence(5'–3')	Desired mutation
Cloning			
Xyn10A-WT	Forward	GCCGCGCGGCAGCATGACCTTAATTGGTATAG	
	Reverse	GCGGCCGCAAGCGTTTATTCTTCTGGCACAAC	
Xyn10A-TM1	Forward	GCCGCGCGGCAGCATGTCTCATCTAATAAAATT	
	Reverse	GCGGCCGCAAGCGTTTATTCTTCTGGCACAAC	
Xyn10A-TM2	Forward	GCCGCGCGGCAGCATGTCTTTGAGAGAGAAATAC	
	Reverse	GCGGCCGCAAGCGTTTATTCTTCTGGCACAAC	
Xyn10B[a]	Forward	GGAATTCCATATGAGCGAAGATTATTATG	
	Reverse	CCGCTCGAGTAAAAAGTCAATTATTCTAAAAAATG	
Point mutation[b]			
TM2-E493A	Forward	GGATGTTGTGAATGCGGCTGTTGAGGATAAAAC	Glu493Ala
	Reverse	TTATCCTCAACAGCCGCATTCACAACATCCC	
TM2-E601A	Forward	GCTTGAAATACAAATAACAGCGCTTGATATATCAGTATTTG	Glu601Ala
TM2-W658A	Forward	CAAAGATGACTATTCAGCGCTTCAAGGTGATTTTC	Trp658Ala
Xyn10B-E139A	Forward	ATGGGATGTGGTAAATGCGGCTATTGATGAAAATG	Glu139Ala
Xyn10B-E247A	Forward	ATTAAGATACATGTCACAGCGATTGATATAAGTGTGGC	Glu247Ala
Xyn10B-E305A	Forward	AGCGATGGACATACAGCGAAAGACAATTTTCC	Trp305Ala

a. Underlined sequences indicate the restriction site.

b. Underlined sequences indicate the substituted codon.
